# DNA methylation suppresses chitin degradation and promotes the wing development by inhibiting Bmara-mediated chitinase expression in the silkworm, *Bombyx mori*

**DOI:** 10.1186/s13072-020-00356-6

**Published:** 2020-09-04

**Authors:** Guanfeng Xu, Yangqin Yi, Hao Lyu, Chengcheng Gong, Qili Feng, Qisheng Song, Xuezhen Peng, Lin Liu, Sichun Zheng

**Affiliations:** 1 Guangdong Provincial Key Laboratory of Insect Developmental Biology and Applied Technology, Institute of Insect Science and Technology, School of Life Sciences, South China Normal University, Guangzhou, 510631 China; 2 Guangzhou Key Laboratory of Insect Development Regulation and Applied Research, Institute of Insect Science and Technology, School of Life Sciences, South China Normal University, Guangzhou, 510631 China; 3 Division of Plant Sciences, College of Agriculture, Food and Natural Resources, University of Missouri, Columbia, MO 65211 USA

**Keywords:** DNA methylation, Chitin degradation, Chitinase, *Bmara*, Transcriptional regulation, Wing development

## Abstract

**Background:**

DNA methylation, as an essential epigenetic modification found in mammals and plants, has been implicated to play an important role in insect reproduction. However, the functional role and the regulatory mechanism of DNA methylation during insect organ or tissue development are far from being clear.

**Results:**

Here, we found that DNA methylation inhibitor (5-aza-dC) treatment in newly molted pupae decreased the chitin content of pupal wing discs and adult wings and resulted in wing deformity of *Bombyx mori*. Transcriptome analysis revealed that the up-regulation of chitinase 10 (*BmCHT10*) gene might be related to the decrease of chitin content induced by 5-aza-dC treatment. Further, the luciferase activity assays demonstrated that DNA methylation suppressed the promoter activity of *BmCHT10* by down-regulating the transcription factor, homeobox protein araucan (*Bmara*). Electrophoretic mobility shift assay, DNA pull-down and chromatin immunoprecipitation demonstrated that Bmara directly bound to the *BmCHT10* promoter. Therefore, DNA methylation is involved in keeping the structural integrity of the silkworm wings from unwanted chitin degradation, as a consequence, it promotes the wing development of *B. mori*.

**Conclusions:**

This study reveals that DNA methylation plays an important role in the wing development of *B. mori*. Our results support that the indirect transcriptional repression of a chitin degradation-related gene *BmCHT10* by DNA methylation is necessary to keep the proper wing development in *B. mori*.

## Background

DNA methylation is an essential epigenetic modification found in eukaryotes [1, 2]. In mammals, about 70% cytosine (C) of the genomic CG context can be methylated by DNA methyltransferases (Dnmts) to generate 5-methylcytosine (5mC) [1, 3]. 5mC is associated with numerous biological processes including cell differentiation [4], transposon silencing [4], genomic imprinting [4, 5], X chromosome inactivation [6], embryonic development [7], and especially gene inactivation [8, 9]. In insects, several functional roles have been revealed for DNA methylation, including reproduction [10–12], memory [13], longevity [14], phenotypic plasticity [15, 16], and social behavior and caste differentiation [17, 18]. However, the role of DNA methylation in insect organ or tissue development remains unclear.

In general, DNA methylation consists of two necessary processes: the establishment of new 5-methylcytosine (5mC) sites mediated by Dnmt3 (de novo DNA methyltransferase) and the maintaining of the existing 5mC mediated by Dnmt1 (maintenance DNA methyltransferase) [1, 3]. Indeed, some insects do possess the Dnmt1/Dnmt3 toolkits as in mammals, for example, in *Apis mellifera* (honey bee), *Nasonia vitripennis* (parasitoid wasp) and *Acyrthosiphon pisum* (pea aphid) [19]. However, some insects, such as *Bombyx mori* (silkworm), *Tribolium castaneum* (red flour beetle) and *Pediculus humanus* (body louse) only have Dnmt1 [19]. Moreover, *Drosophila melanogaster* even lost the Dnmt1/Dnmt3 toolkits [19, 20]. Although the existence of 5mC DNA methylation in *Drosophila* has been disputed, there is an evidence showing that 5mC could be deposited in the promoter region of *Drosophila* gene *DmSpok*, and the treatment of DNA methylation inhibitor 5-aza-dC, also named as decitabine, could eliminate 5mC in the region and enhanced the transcriptional activity of *DmSpok* [21]. However, how the gene is methylated in *Drosophila* is unclear, and how 5-aza-dC works in *Drosophila* also needs to be investigated. Interestingly, the *B. mori* possesses only one DNA methyltransferase BmDnmt1, but exhibits 5mC DNA methylation [22], suggesting that BmDnmt1 may have dual functions of both de novo methylation and maintenance methylation [20, 23]. Our previous work showed that *BmDnmt1* RNAi enhanced the transcription of a chitin synthase gene *BmCHSA*-*2b*, and 5-aza-dC treatment obtained a similar effect as RNAi [24], indicating that *BmDnmt1* play a regulatory role in gene transcription. However, whether BmDnmt1 has dual functions of both de novo methylation and maintenance methylation need to be investigated.

To date, most studies on insect DNA methylation are based primarily on the insect methylome sequencing and the manipulation of functional *Dnmt* genes. By means of whole-genome bisulfite sequencing (WGBS-seq), a single-base resolution of methylome has so far been acquired in several insect species, including *A. mellifera* [25], *Bombus terrestris* (bumblebee) [26], *Camponotus floridanus* (ant) [17], *Harpegnathos saltator* (ant) [17], *Solenopsis invicta* (ant) [27], *N. vitripennis* [28] and *B. mori* [22]. These methylome studies have offered us valuable information of DNA methylation in insects. Meanwhile,functional studies based on *Dnmt1/Dnmt3* RNAi had also extended our understanding of the functional role of insect methylation. For instance, we now know that *Dnmt1* plays roles in egg production and embryo viability in *O. fasciatus* [12] and gene expression in *B. mori* [24], and that *Dnmt3* functions as regulator in gene expression and alternative splicing in *A. mellifera* [29]. However, *Dnmt1/Dnmt3* RNAi may not be suitable for all insect species, especially those with RNAi insensitivity. Other approaches, like CRISPR–Cas9 and DNA methylation inhibitor, were used for the functional study of DNA methylation. CRISPR–Cas9 system is thought to be a manageable tool to create *Dnmt1/Dnmt3* mutants in insects, especially those non-model insects [23, 30]. 5-Aza-dC (5-aza-2′-deoxycytidine), a nucleoside analogue of cytosine that can disturb DNA methylation, has been commonly used as a de-methylation agent, not only in clinical practice like the treatments of chronic myelomonocytic leukemia, refractory anemia, myelodysplastic syndrome and acute myeloid leukemia [31, 32], but also in scientific researches, such as sexual development in zebrafish [33], fruit ripening in tomato [34], and gene expression in mammals and insects [21, 35, 36].

In mammals, methylation frequently occurs in the promoters and results in a reduction in gene expression by blocking the binding of transcription factors to the promoter [9]. While in insects, most methylated cytosines occur over gene bodies, and methylation levels in gene bodies are positively correlated with gene expression levels [22, 28]. Whether and how intragenic DNA methylation enhances insect gene expression is unknown. Recently, DNA methylation on gene promoter regions in *Drosophila* and *B. mori* was reported [21, 24]. Dmkrh1 of juvenile hormone regulatory pathway promotes the hypermethylation of *DmSpok* promoter region, reducing the transcriptional activity of *DmSpok* gene [21]. Since *Drosophila* does not have Dnmt1 and Dnmt3, how *DmSpok* gene is methylated is still unclear. In *B. mori*, the de-methylation of promoter allowed the binding of stage-specific transcription factor Deaf1 to the promoter of chitin synthase *BmCHSA*-*2b* and enhanced gene expression at the middle stages of pupal wing disc development [24]. Nevertheless, how DNA methylation regulates gene expression to impact *B. mori* wing development is not well understood.

Insect wings play important roles in flight, orientation, protection, communication, and courtship of *Lepidopteran* insect [37]. *B. mori* is a model insect of *Lepidoptera*. Although *B. mori* adult cannot fly, the male needs to flap its wings to copulate [38]. Therefore, the wing is important for *B. mori* reproduction. The life cycle of lepidopteran insect includes four stages: egg, larva, pupa and adult [37]. From the late larval stage, wing discs of lepidopteran insects arose as outgrowth of body wall (i.e., epidermal cells and procuticle) to generate the pupal wing discs. When larvae develop into pupae, the arisen wing discs gradually become a sandwich with two epicuticular layers filled with hemolymph, nerves and tracheas [39]. Remarkably, chitin, a polymer of *N*-acetylglucosamine, is an essential component of the exoskeleton of the wing epicuticular layers. Combining with cuticle proteins, chitin comprises the main structural materials of insect wings [40]. During pupal stages, *B. mori* wing discs continue growth and the chitin content continue to increase [41]. The chitin content in the wing is a balance of chitin synthesis and chitin degradation, which are catalyzed by chitin synthases and chitinases [40], respectively. Our previous study demonstrated that DNA methylation regulates the expression of chitin synthetase gene *BmCHSA*-*2b* in the *B. mori* pupal wing discs [24]. However, the question whether DNA methylation is engaged in *B. mori* wing development by affecting chitin content has not been addressed.

In this study, we report a study primarily based on DNA methylation inhibitor (5-aza-dC) treatment in *B. mori*. We found that DNA methylation regulates the development of *B. mori* pupal wing disc by inhibiting the expression of chitinase gene *BmCHT10* via transcription factor Bmara in the pupal wing disc, leading to inhibitory effect of chitin degradation in the pupal wing disc. Our results offer a deeper insight into the impact of DNA methylation on *B. mori* wing development.

## Results

### DNA methylation affects wing development and chitin formation

**Fig. 1:**
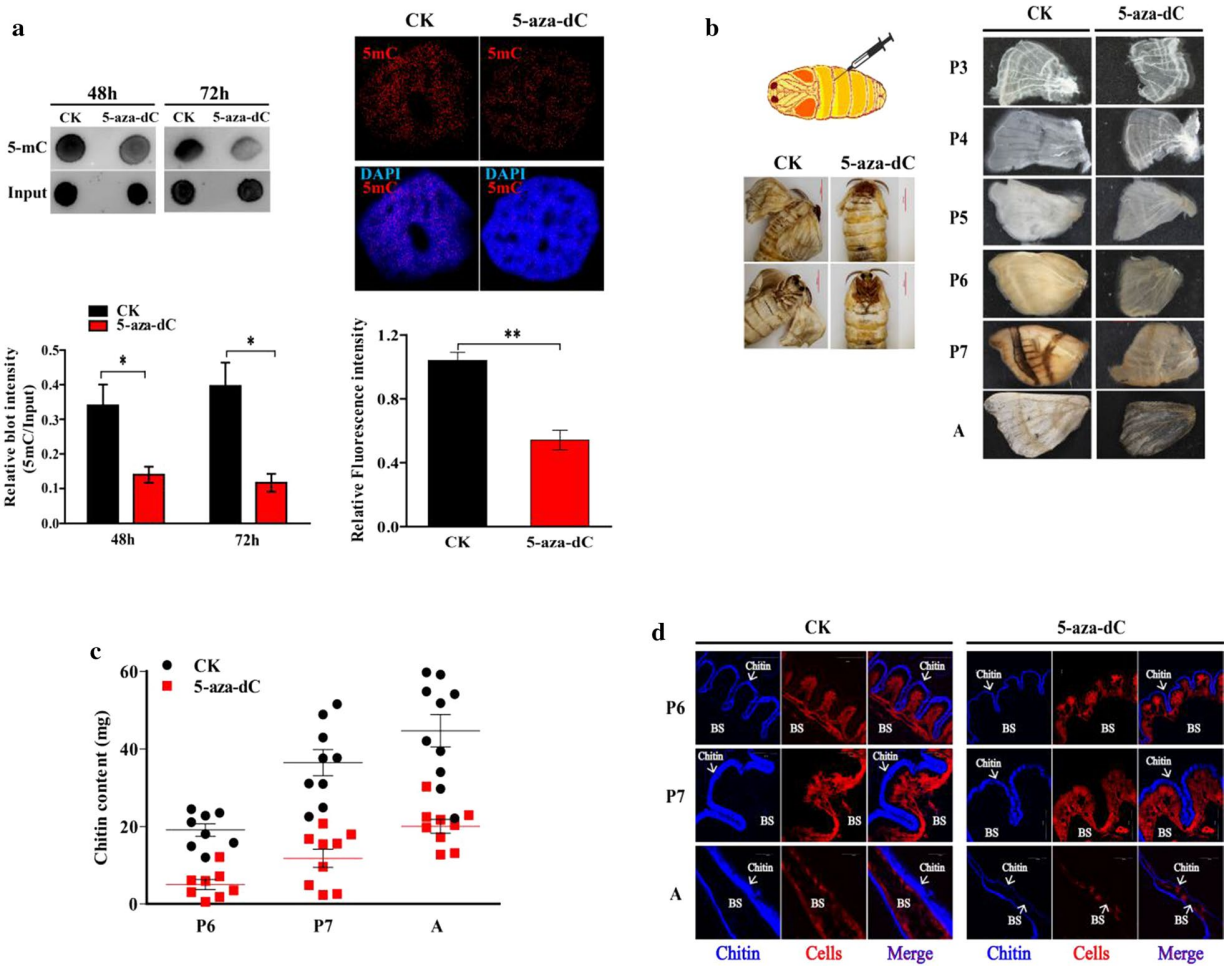
DNA methylation mediates the change of chitin degradation in *Bombyx mori* wing. **a** Dot blot analysis (left) and immunofluorescent staining (right) of DNA methylation levels 48 h after treatment of newly molted pupae with 5-aza-dC. Dot blot was conducted using 5mC antibody (top left) and then the quantitative analysis of relative blot intensity (5mC/Input) was calculated automatically using ImageJ software (bottom left). 5mCs were stained with secondary antibody (right, red) and the nuclei were stained with DAPI (right, blue). The relative fluorescence intensity of 5mCs was calculated using ImageJ software (bottom right). The images were captured by a laser confocal microscope. Scale bar: 40 μm. 5mC: 5-methylcytosine. DAPI: 4′, 6-diamidino-2-phenylindole. **b** A newly molted pupa (P0 stage) was injected with 20 μg 5-aza-dC- or control ddH_2_O. Phenotype changes at adult stage were captured by stereomicroscope. Scale bar: 5000 μm (left). Phenotype differences between the wing discs of 5-aza-dC- or ddH_2_O-treated silkworm at different pupal developmental stages were captured by stereomicroscope. Scale bar: 2000 μm. Pn: n-day-old of pupae. A: Adult. **c** Chitin from the pupal wing discs or adult wings of 5-aza-dC- or ddH_2_O-treated silkworm at P6-A stages were extracted for chitin content assays using spectrophotometry. **d** Chitin staining of the crosscut wings of 5-aza-dC- or ddH_2_O- treated silkworm at P6-A stages. Chitin was stained with Fluorescent Brightener 28 (blue) and the nuclei were stained with propidium iodide (red). The images were captured by a laser confocal microscope. Scale bar: 40 μm. Each data point is the mean ± SE of three independent assays. For the *t* test: *p* < 0.05 (*) or *p* < 0.01(**)

In order to investigate whether and how DNA methylation impacts the wing development, we used DNA methylation inhibitor 5-aza-dC to treat *B. mori* since *Bmdnmt1* RNAi did not work in pupae and 5-aza-dC has been shown to effectively inhibit DNA methylation in *B. mori* [24]. Dot blot and immunofluorescent staining with 5mC antibody showed that injection of 5-aza-dC in newly molted pupae (P0) induced the de-methylation of 5mC in the genome (Fig. 1a), indicating that 5-aza-dC can be used for the study of 5mC function in silkworm. After the inhibitor treatment, all the 5-aza-dC-treated wings became smaller, thinner and with less hair starting from 3-day-old pupa (P3) to adult (A) compared to control groups (Fig. 1b). These results suggest that DNA methylation is involved in the regulation of silkworm wing development. Further, the content of chitin, the main component of wing skeleton including wing procuticle, vein and hair, was determined. The result showed that the chitin content in the silkworm wings was significantly decreased by 5-aza-dC treatment (Fig. 1c). The staining of the wing slices showed that the chitin layer (blue) became thinner and two procuticle layers became closer after 5-aza-dC treatment (Fig. 1d). These results suggest that DNA de-methylation can damage wing development by inhibiting the chitin formation in the pupal wing discs of *B. mori*.

### DNA methylation affects chitin formation by inhibiting the expression of *BmCHT10*

**Fig. 2:**
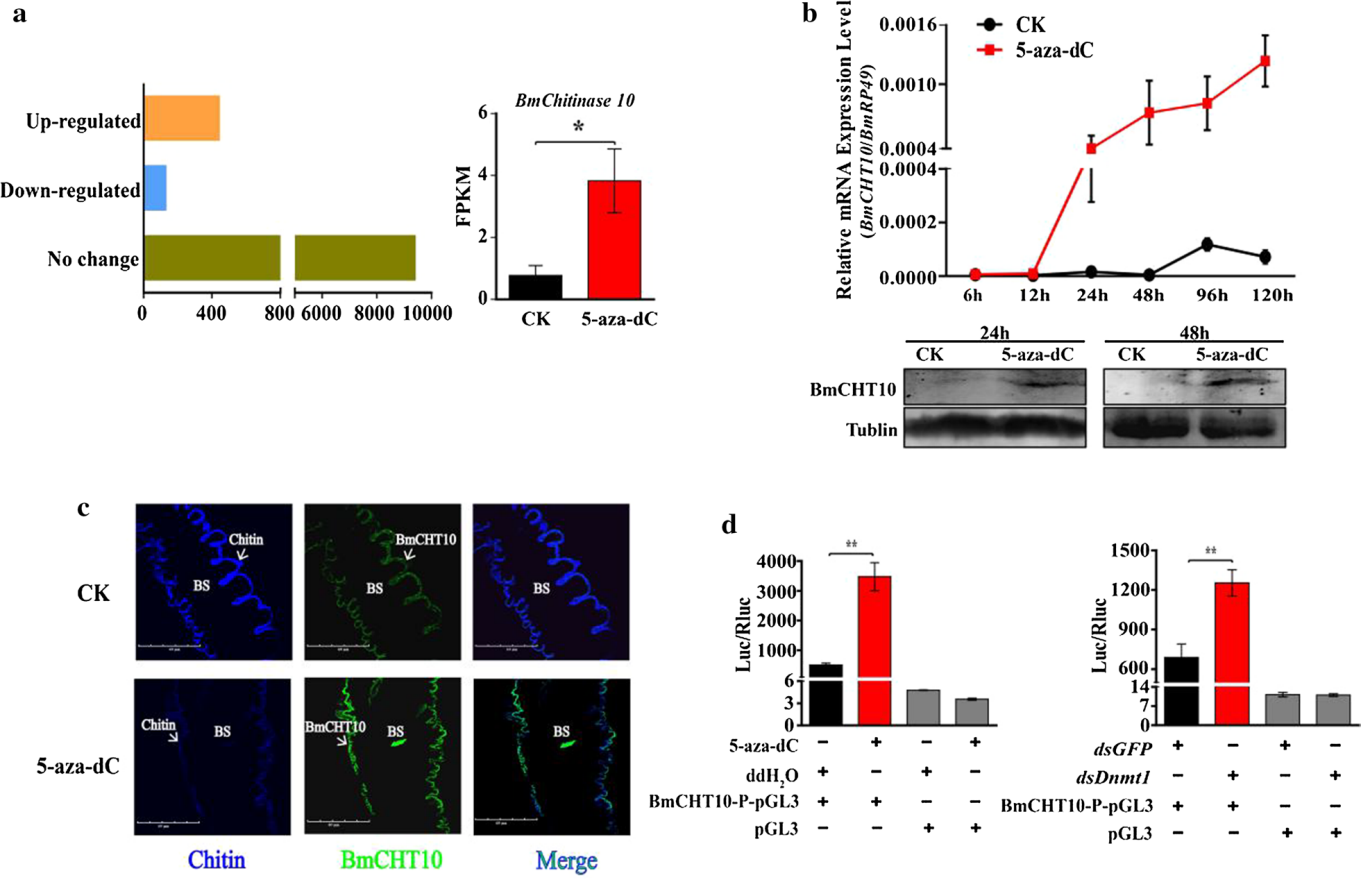
DNA methylation affects chitin formation in the pupal wing discs of *B. mori* via *BmCHT10*. **a** Transcriptomic analysis of gene transcriptional levels in 5-aza-dC-treated wing discs of 3-day-old pupae. **b** Effects of 5-aza-dC treatment on the mRNA (top) and protein (bottom) levels of *BmCHT10* at pupal stages. **c** Immunohistochemistry analyses of the effects of 5-aza-dC treatment on chitin (blue) formation and BmCHT10 protein (green) levels in the 3-day-old pupal wing disc. Scale bar: 60 μm. **d** Effects of 5-aza-dC treatment (left) or *Dnmt1* RNAi (right) on the luciferase activity of the − 1100 to − 1 nt fragment of *BmCHT10* promoter in the *Bm*12 cells. One microgram 5-aza-dC or *dsBmDnmt1* with the vector including − 1100 to − 1 nt fragment was transfected. ddH_2_0 or *dsgfp* was used as a control. Each data point is the mean ± SE of three independent assays. For the *t* test: *p* < 0.05 (*) or *p* < 0.01(**)

To investigate how DNA de-methylation caused by 5-aza-dC treatment affects the silkworm wing development and chitin formation, we performed transcriptome analysis. 5-Aza-dC was injected at P0 stage, silkworm wing discs were collected for RNA-seq 48 h later. The sequence result showed there were a total 585 differentially expressed genes (DEGs) in 5-aza-dC-treated samples compared to control, including 449 up- and 136 down-regulated genes (Fig. 2a). Among up-regulated genes, most of them encode cuticle proteins (Additional file 1: Table S1), while three code for proteins related to chitinases (GO:0004568), which catalyze chitin degradation. Three up-regulated chitinase genes included one encoding chitinase-related protein1 (ChiR1) (GenBank accession no. 692403) and two annotated as chitinase 10 (CHT10) encoding gene (GenBank accession no. 101736080) (Additional file 1: Table S1). BmChiR1 is reported to involve the chitin catabolic process, however, it has high similarities to arthropod chitinases but lacks the active site of glutamate for catalytic activity, suggesting that BmChiR1 protein has no chitinolytic activity [42], while Chitinase 10 protein plays a vital role in chitin degradation [40]. Therefore, the up-regulation of *BmCHT10* by 5-aza-dC treatment may be the main factor that causes chitin degradation in the *B. mori* pupal wing disc. Since 5-aza-dC treatment resulted in the change of size, thickness and hair of wings, which are mostly related to chitin content, we focused on studying the relationship between 5mC and *BmCHT10*.

*CHT10* has been previously reported to involve the chitin degradation and insect molting process in *Locusta migratoria* [43], *T. castaneum* [40] and *Nilaparvata lugens* [44]. Comparing the conserved domains of *B. mori* CHT10 protein with CHT10 of these three insects revealed that CHT10 in all these species contained glycoside hydrolase family 18 (GH18 family) catalytic domain (Glyco_18) (SMART ID: SM00636) and chitin-binding domain type 2 (ChtBD2) (SMART ID: SM000494). LmCHT10 and TcCHT10 have five copies of the Glyco_18 domains, while NlCHT10 and BmCHT10 have only two copies (Additional file 1: Figure S1). It is reported that Glyco_18 domain has four conserved regions (CRs) [44], and the CR_II region (DWEYP) is essential for the characterization of a putative chitinase while its residue E (Glu) is essential for catalytic activity [45]. Therefore, we analyzed the sequences of the 14 Glyco_18 domains from LmCHT10, TcCHT10, NlCHT10 and BmCHT10. We found that all the four regions in the two BmCHT10 Glyco_18 domains are highly conserved compared to the others, and the E (Glu) residue in the CR_II region is fully conserved in all the Glyco_18 domains of four insect CHT10s (Additional file 1: Figure S1), suggesting that insect CHT10s may have the similar function.

Further, to confirm whether *BmCHT10* expression is regulated by 5mC, we analyzed the change of *BmCHT10* expression after 5-aza-dC treatment. We found that the mRNA and protein levels of *BmCHT10* in the pupal wing discs were significantly increased from 24 h to 120 h post 5-aza-dC treatment compared to the untreated group (Fig. 2b). Immunohistochemistry confirmed that BmCHT10 proteins were co-localized with chitins in the pupal wing disc, and BmCHT10 proteins in the pupal wing disc were increased post 5-aza-dC treatment (Fig. 2c). To investigate how 5mC regulates the expression of *BmCHT10*, we analyzed the transcriptional activity of the promoter. In the *Bm*12 cells, both 5-aza-dC treatment (Fig. 2c; left panel) and *BmDnmt1* RNAi (Fig. 2c; right panel and Additional file 1: Figure S2) showed that the transcriptional activity of the *BmCHT10* promoter (− 1100 to − 1 nt) was significantly enhanced by DNA de-methylation compared to control (without *BmCHT10* promoter). These findings together suggest that DNA methylation may affect chitin content in the pupal wing disc via *BmCHT10*.

### DNA methylation regulates the transcription of *BmCHT10* via the promoter

**Fig. 3:**
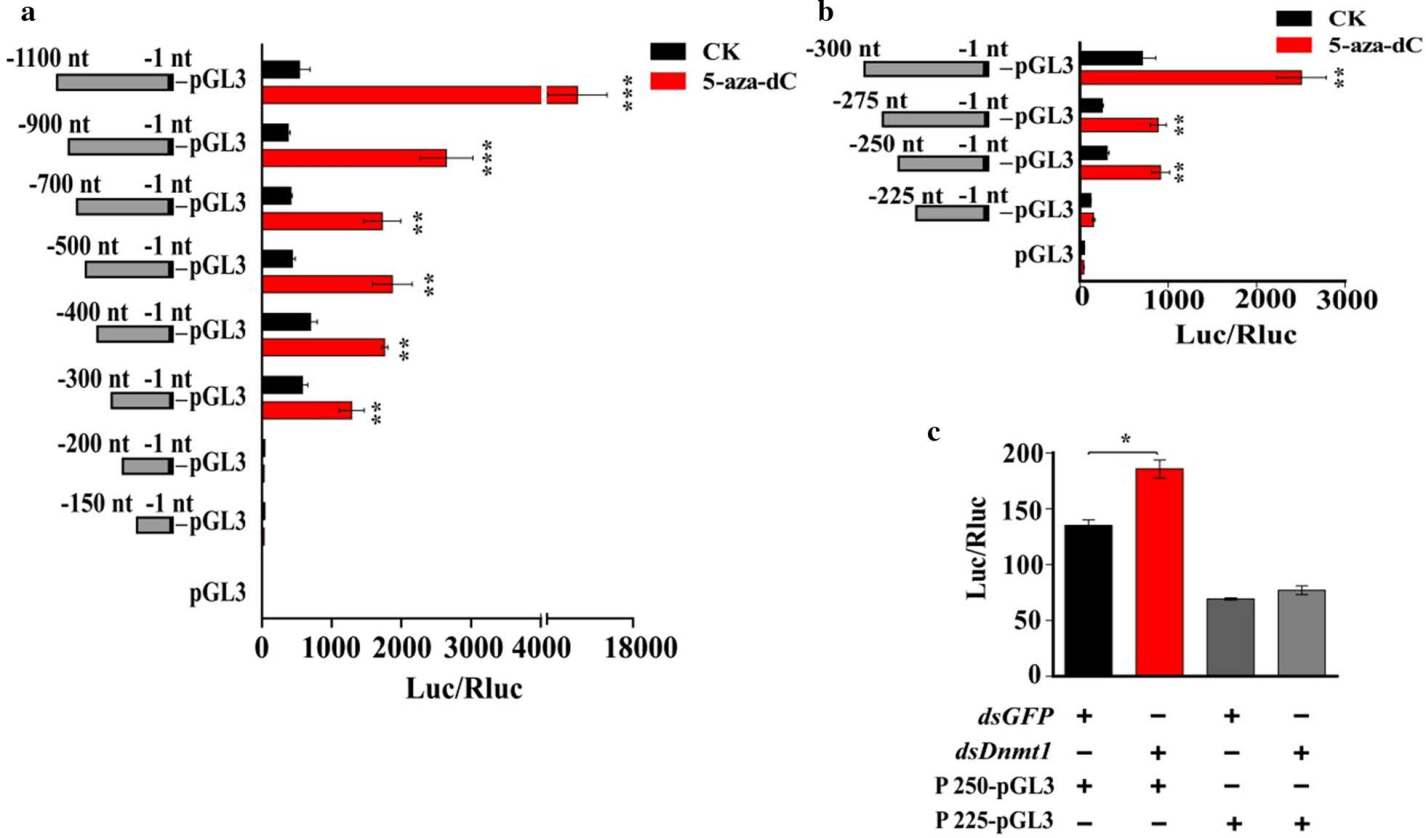
DNA methylation regulates the transcriptional activity of *BmCHT10* via its promoter. **a** Effects of 5-aza-dC treatment on the luciferase activities of the different truncated fragments of − 1100 to − 1 nt region of the *BmCHT10* promoter in *Bm*12 cells. **b** Effects of 5-aza-dC treatment on the luciferase activities of the different truncated fragments of − 300 to − 1 nt region of the *BmCHT10* promoter in *Bm*12 cells: − 300 to − 1 nt, − 275 to − 1 nt, − 250 to − 1 nt and − 225 to − 1 nt promoter fragments. The cells were transfected with the luciferase vector including different lengths of promoter fragments and added with 1 μL of 1 μg/μL 5-aza-dC or ddH_2_0 (as control), respectively. **c** Effects of *BmDnmt1* RNAi on the luciferase activity of the − 250 to − 1 nt promoter region of *BmCHT10* in the *Bm*12 cells. *dsgfp* or *dsBmDnmt1* was co-transfected with the vector including − 250 to − 1 nt promoter fragment for the determination of luciferase activity. Each data point is the mean ± SE of three independent assays. For the *t* test: *p *< 0.05 (*), *p *< 0.01(**) or *p *< 0.001(***)

To determine which region in the *BmCHT10* promoter is responsive to 5-aza-dC treatment, luciferase activity assays in *Bm*12 cells were performed. We inserted 1100-bp-length *BmCHT10* promoter or its truncated fragments into the pGL3-Basic vector. The luciferase activity results showed that the − 300 to − 200 nt, − 900 to − 700 nt and − 1100 to − 900 nt regions of the *BmCHT10* promoter could significantly respond to the 5-aza-dC treatment, especially the − 300 to − 200 nt fragment (Fig. 3a). Subsequently, the promoter region of − 300 to − 200 nt was truncated to narrow the scope, and the results revealed that 5-aza-dC treatment significantly induced the transcriptional activity of the promoter region at − 250 to − 225 nt (TTCAGTCCACGGCTGTCTTTCAACACGGA) (Fig. 3b), suggesting that the − 250 to − 225 nt region is the most responsive region to 5-aza-dC treatment. Similarly, the luciferase activity of − 250 to − 225 nt region was significantly increased after *BmDnmt* RNAi (Fig. 3c). These results suggest that DNA methylation may suppress the transcription of *BmCHT10* via the promoter, and − 250 to − 225 nt is the most responsive region.

### DNA methylation regulates the transcriptional activity of the *BmCHT10* promoter by inhibiting the expression of *Bmara*

**Fig. 4:**
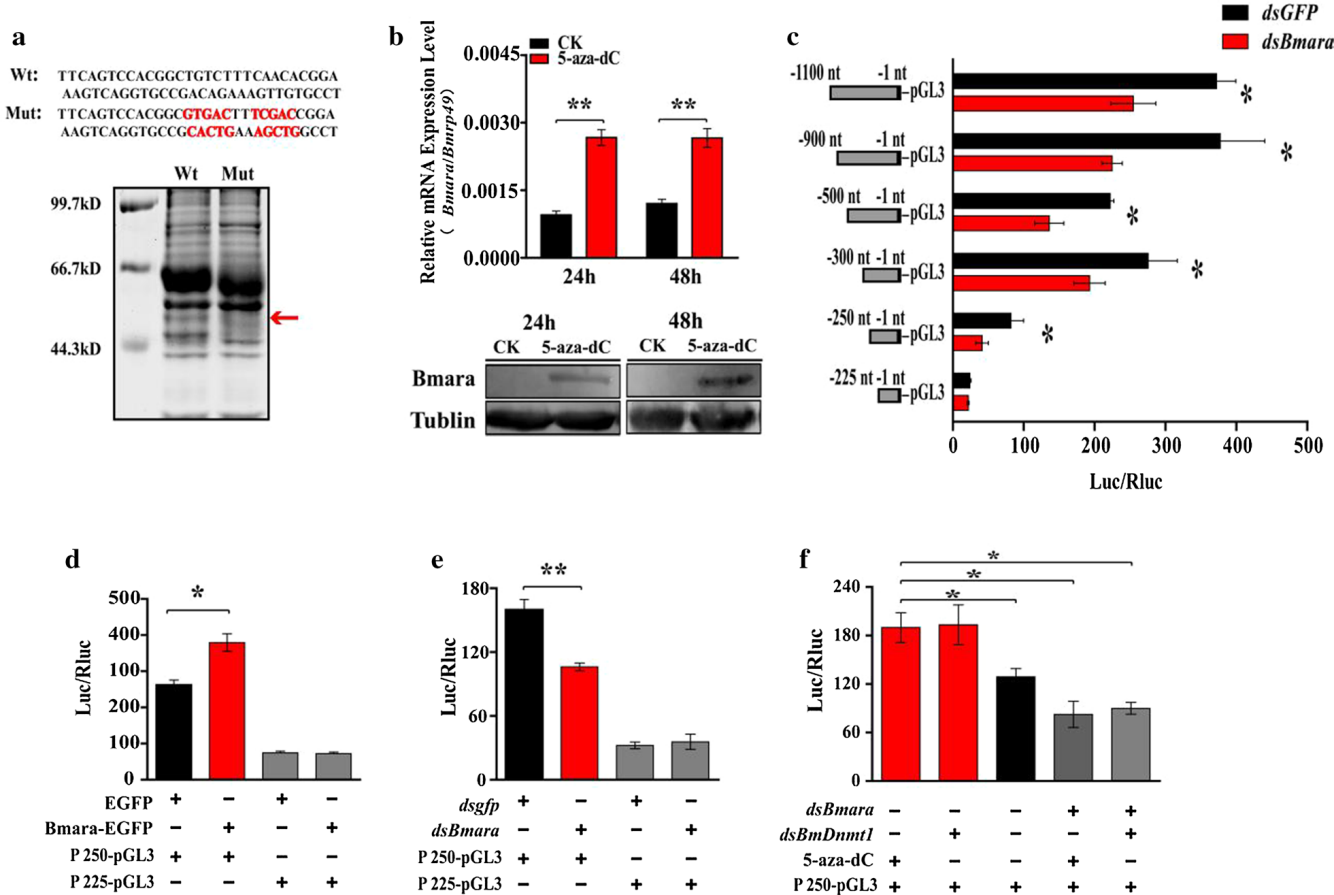
DNA methylation regulates the expression of *BmCHT10* via Bmara. **a**. DNA pull-down experiment with the wild type or mutated − 250 to − 225 nt fragment of the *BmCHT10* promoter (DNA probe) and the nuclear proteins isolated from the wing disc of 3-day-old pupae. **b** Effects of 5-aza-dC treatment on the mRNA (left) and protein (right) levels of Bmara at pupal stages. **c** Effects of *Bmara* RNAi on the luciferase activities of the different promoter truncates of *BmCHT10* in *Bm*12 cells: − 1100 to − 1 nt, − 900 to − 1 nt, − 500 to − 1 nt, − 300 to − 1 nt, − 250 to − 1 nt and − 225 to − 1 nt promoter fragments. **d** Effects of Bmara overexpression on the reporter luciferase activity under the control of the − 250 to − 225 nt fragment of *BmCHT10* promoter in the *Bm*12 cells. **e** Effects of *Bmara* RNAi on the reporter luciferase activity under the control of the − 250 to − 225 nt fragment of *BmCHT10* promoter in the *Bm*12 cells. **f** Effects of 5-aza-dC treatment or *BmDnmt1* RNAi on the luciferase activity of − 250 to − 225 nt promoter fragment after *Bmara* RNAi. Each data point is the mean ± SE of three independent assays. For the *t* test: *p *< 0.05 (*) or *p *< 0.01(**)

To investigate whether 5mC presented in the − 250 to − 225 nt region of the *BmCHT10* promoter directly regulates *BmCHT10* expression, we performed the bisulfite sequencing PCR (BSP) to check the single-base-resolution DNA methylation pattern at the − 250 to − 225 nt region. The result showed that cytosines located in the − 250 to − 225 nt region were not methylated (Additional file 1: Table S2). Thus, we proposed that the DNA methylation may regulate the transcription of *BmCHT10* through its upstream transcription factors. To identify which protein may regulate the transcriptional activity of the − 250 to − 225 nt region, the *cis*-regulatory elements (CRE) were predicated using JASPAR software [46]. Five kinds of CREs present in the − 250 to − 225 nt region, but only ara CRE appears in all 5-aza-dC treatment-responsive promoter regions (Additional file 1: Table S3 and S4). To further identify the transcription factor, a pull-down experiment was conducted. The oligonucleotide fragment of − 250 to − 225 nt was labeled with biotin and linked to the streptavidin-coated beads. The complex was then incubated with the nuclear proteins isolated from *Bm*12 cells. The proteins that bound to the biotin-labeled probe were separated on a SDS-PAGE gel. The results showed that two protein bands (approx. 50 and 45 kDa) disappeared after the core sequence of ara CRE in the probe was mutated (Fig. 4a), suggesting these proteins might bind to the oligo-probe.

To identify what proteins may bind to CRE in the − 250 to − 225 nt region, we analyzed the potential binding proteins predicted by the JASPAR software [46]. Among the potential binding proteins with the − 250 to − 225 nt region, *B. mori* homeobox protein araucan (Bmara) (51.13 kDa) (GenBank accession no. 101745000) was consistent with the above-mentioned 50-kDa differential protein band (Additional file 1: Table S3), and protein caupolican or homothorax was consistent with the 45 kDa band in the gel for the pull-down assay. However, RNAi results of these three genes showed that only Bmara regulated *BmCHT10* transcription (Additional file 1: Figure S3). Therefore, we investigated whether Bmara regulates *BmCHT10* expression. In the 5-aza-dC-treated pupal wing discs, the mRNA and protein levels of *Bmara* were increased significantly compared with the control (Fig. 4b), suggesting that DNA methylation inhibits the expression of *Bmara*. To determine whether Bmara regulates the transcriptional activity of *BmCHT10*, luciferase activity assay was conducted. After *Bmara* RNAi, the luciferase activities of promoter fragments including − 250 to − 225 nt were inhibited (Fig. 4c). Further, the regulation of Bmara to the transcriptional activity of − 250 to − 225 nt fragment was confirmed by Bmara overexpression. The Bmara-EGFP vector (Additional file 1: Figure S4) was co-transfected with the − 250 to − 1 nt or − 225 to − 1 nt fragment-containing luciferase vector into the *Bm*12 cells. The result showed that the luciferase activity of − 250 to − 1 nt, but not − 225 to − 1 nt fragment, of the *BmCHT10* promoter was significantly increased by Bmara overexpression (Fig. 4d). On the contrary, *dsBmara* (Additional file 1: Figure S5) was co-transfected with the − 250 to − 1 nt or − 225 to − 1 fragment-containing luciferase vector, only the luciferase activity of − 250 to − 1 nt was decreased (Fig. 4e). These results suggest that Bmara can enhance the luciferase activity of − 250 to − 225 nt fragment. Furthermore, after *Bmara* was knocked down by RNAi, the luciferase activity of − 250 to − 225 nt fragment could not be induced by 5-aza-dC treatment or *BmDnmt1* RNAi (Fig. 4f). These results suggest that DNA methylation suppresses the expression of *Bmara*, leading to the inhibition of transcriptional activity of the *BmCHT10* promoter.

### Bmara directly binds to the − 250 to − 225 nt fragment of the *BmCHT10* promoter

**Fig. 5:**
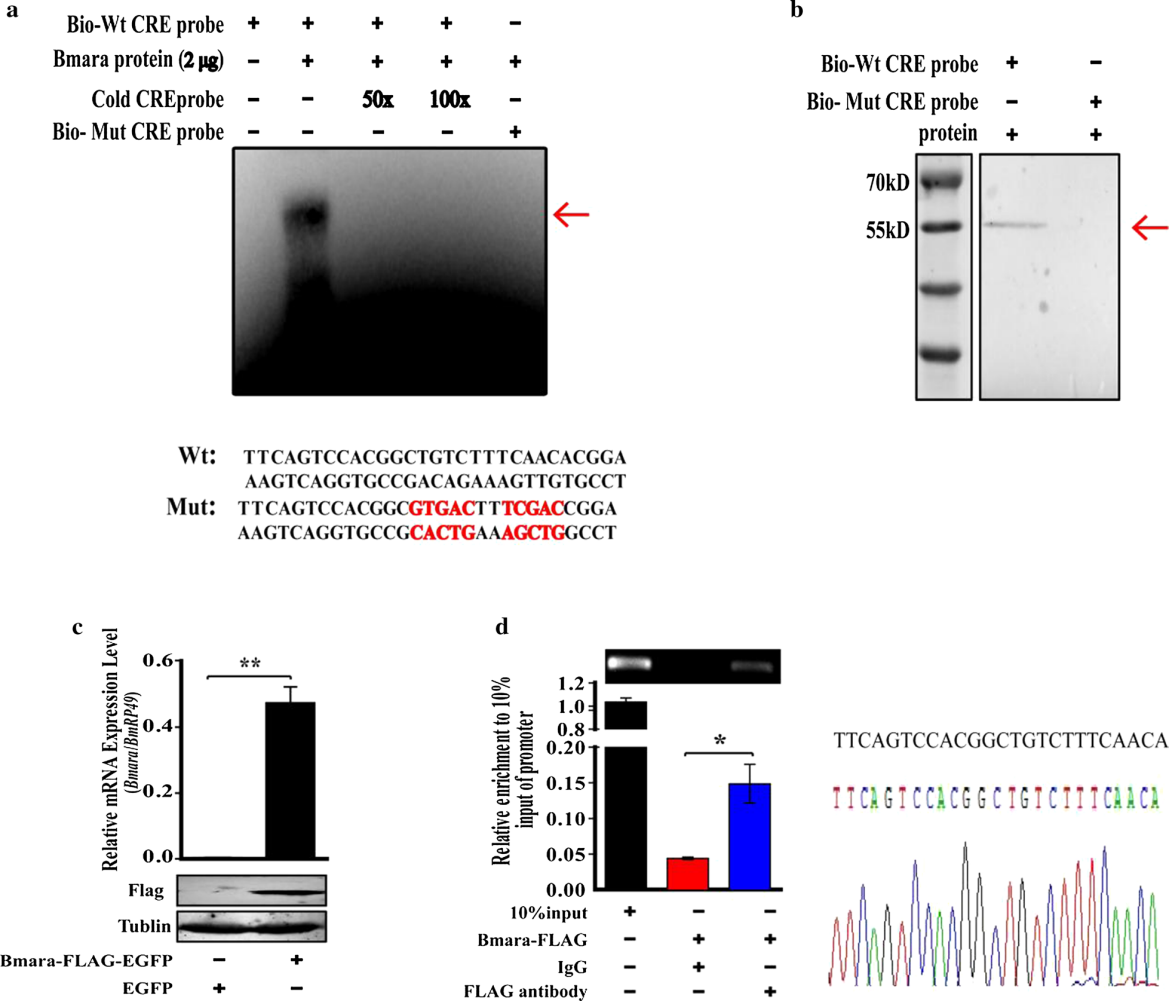
Analysis of the binding of Bmara with the − 250 to − 225 nt fragment of *BmCHT10* promoter. **a** Electrophoretic mobility shift assay (EMSA) of the binding of the purified Bmara proteins to the biotin-labeled probe of wild type − 250 to − 221 nt fragment. The cold probe is the unlabeled probe. **b** DNA pull-down experiment with the wild type or mutated − 250 to − 225 nt fragment and the nuclear proteins from the *Bm*12 cells that were transfected with Bmara-FLAG overexpression vector. The proteins that bound to the − 250 to − 225 nt probe in the supernatant were visualized by Western blot with the antibody against FLAG. **c** qRT-PCR and Western blot analyses showed that the mRNA and protein levels of Bmara in the *Bm*12 cells were increased when Bmara-FLAG was overexpressed. **d** The chromatin immunoprecipitation targets were detected by RT-PCR and qRT-PCR (left) in the *Bm*12 cells. The enriched RT-PCR product of the ChIP assay was sequenced and the sequence was aligned with the − 250 to − 225 nt fragment (right). Each data point is the mean ± SE of three independent assays. For the *t* test: *p* < 0.05 (*) or *p* < 0.01(**)

Since a protein–DNA interaction has been found between the Bmara protein and the − 250 to − 225 nt fragment of the *BmCHT10* promoter, we wondered if Bmara proteins directly bound to the *BmCHT10* promoter. The ORF of the *Bmara* was cloned and a recombinant protein was expressed in *E. coli* and purified for electrophoretic mobility shift assay (EMSA). The result showed that the recombinant Bmara proteins specifically bound to the biotin-labeled probe, the − 250 to − 225 nt fragment. The binding could be competed off by the 50× and 100× unlabeled probe. When the probe was mutated, the binding was lost (Fig. 5a). Similarly, the result from DNA pull-down assay showed that Bmara bound to the biotin-labeled − 250 to − 225 nt probe (Fig. 5b). Further, chromatin immunoprecipitation (ChIP) was also performed. The *Bm*12 cells were transfected with the recombinant plasmid Bmara-FLAG overexpression vector, and Bmara proteins in the cells were confirmed by Western blot (Fig. 5c). In the Bmara-FLAG overexpressed cells, the anti-FLAG antibodies, but not the control IgG, precipitated and enriched the − 250 to − 225 nt fragment of the *BmCHT10* promoter (Fig. 5d; left panel). The enriched − 250 to − 225 nt fragment was amplified by PCR and its sequence was confirmed by DNA sequencing (Fig. 5d; right panel). Taken together, the results from EMSA, DNA pull-down and ChIP demonstrated that the Bmara protein directly bound to the − 250 to − 225 nt fragment of the *BmCHT10* promoter.

## Discussion

In this study, we report several findings that offer valuable insights into the functional roles of DNA methylation in wing development of *B. mori*. First, it is the first experimental study showing that DNA methylation affects the wing development of *B. mori*. Second, we demonstrated that 5mC inhibitor treatment significantly decreased the chitin content of silkworm wing. Third, we found that 5mC inhibitor treatment caused the up-regulation of *BmCHT10*, a vital chitin degradation-responsible enzyme in insects [40]. Moreover, we found that the *BmCHT10* transcription was activated by Bmara, which was up-regulated by 5mC inhibitor treatment. Therefore, we reveal that DNA methylation-ara-CHT10 pathway affects the *B. mori* wing development.

DNA methylation plays important functional roles in insects [10–18]. However, the effect of DNA methylation on insect wing development is unclear. In this study, we found that DNA methylation affected *B. mori* wing development using DNA methylation inhibitor 5-aza-dC (Fig. 1a). Recently, Dnmt inhibitor has been considered as a potential tactic for functional study of DNA methylation in insects [23]. In this study, injection of 5-aza-dC into newly molted pupae of *B. mori* resulted in the abnormal wing appeared from 3-day-old pupae to adults (Fig. 1). This result indicates that DNA methylation plays a role in *B. mori* wing development. 5-Aza-dC is an effective de-methylation drug which inhibits the deposition of 5mC and blocks DNA methylation [31–33]. Our previous result showed that 5-aza-dC treatment inhibited the methyltransferase activity in the *B. mori* cell line [24]. In this study, we demonstrated that the genome methylation level of *B. mori* pupal wing discs decreased after 5-aza-dC treatment (Fig. 1a). These results suggest that 5-aza-dC treatment eliminates methylation through inhibiting methyltransferase activity in *B. mori*. However, the functional mechanism of 5-aza-dC and dual functions of the de novo methylation and maintenance of BmDmnt1 *in B. mori* deserve further investigation.

We discovered that de-methylation resulted in the decrease of chitin content in *B. mori* wings. Chitin, the main component of two epicuticular layers, composes the major structural component to hold the whole insect wings [39, 40]. The last step of chitin synthesis pathway is catalyzed by chitin synthases, while the degradation is catalyzed by chitinases [40]. In the present study, our results of chitin staining and chitin content assay showed that 5-aza-dC applied in newly molted pupae was capable of undermining the procuticle layers in the sandwich of silkworm wings (Fig. 1d), and significantly decreased the chitin content in silkworm wings (Fig. 1c). Therefore, our results reveal a biological significance of 5mC in wing development by inhibiting chitin degradation. Based on the transcriptomic data of 5-aza-dC-treated 3-day-old silkworm pupal wing discs, we found chitinase *BmCHT10* was up-regulated by 5-aza-dC treatment as determined by qRT-PCR, Western blot and promoter activity determination (Fig. 2b, c). However, we could not find any genes of the chitin synthesis pathway in the transcriptomic data. These results suggest that 5-aza-dC injected into newly molted pupae enhances chitinase expression rather than inhibits the chitin synthase to decrease chitin content in the early pupal wing disc.

*Bombyx mori* wing continues to grow during pupal stages and the chitin content in the pupal wing disc continues to increase. Epidermal chitin synthase 2a (*BmCHSA*-*2a*) is responsible for catalyzing chitin synthesis at the early stages, while *BmCHSA*-*2b* is responsible for the middle stages [41]. Higher levels of DNA methylation at the early stages inhibit *BmCHSA*-*2b* expression, while lower levels of DNA methylation allow *BmCHSA*-*2b* expression [24]. DNA methylation does not affect *BmCHSA*-*2a* expression [24]. In this study, our results indicate that DNA methylation plays a role in pupal wing disc development by inhibiting chitinase *CHT10* expression. Therefore, DNA methylation involves the regulation of both chitin synthesis and degradation. The *BmCHT10*, coding for a key hydrolytic enzyme Chitinase10, was significantly up-regulated in the 5-aza-dC-treated pupal wing discs (Fig. 2). Its homologous proteins are implicated to chitin degradation in other insects as well [40, 43, 44]. *T. castaneum CHT10* RNAi resulted in molting failure and death of larvae and pupae [47]. The analysis of amino acid domains showed that CHT10 proteins in diverse insect species are conservative, suggesting that the similar function of CHT10 in insects (Additional file 1: Figure S1). Therefore, at the early pupal stages with higher DNA methylation levels, the inhibition of chitinase expression by DNA methylation and the up-regulation of *BmCHSA*-*2a* ensure chitin synthesis. At the middle of pupal stages with lower DNA methylation levels, promoter de-methylation allows stage-specific transcription factor Deaf1 to bind the *BmCHSA*-*2b* promoter and activate gene expression [24], leading to continuous chitin synthesis and wing discs growth. The stage-dependent dynamic change of DNA methylation affects pupal wing disc development by regulating chitin synthase and inhibiting chitinase expression at specific development stages to ensure the stable chitin accumulation. Besides *BmCHT10*, our results showed that DNA methylation at the early stages also inhibited other unwanted gene expression, such as redundant cuticle proteins (Additional file 1: Table S1), to ensure accurate wing development. The inhibition of development-related genes like *BmCHT10* at the early stages may explain why the wing phenotype can be obtained when newly pupae were treated with DNA methylation inhibitor.

In this study, we reveal that DNA methylation inhibits the expression of transcription factor araucan (*ara*), leading to the inhibition of *BmCHT10* expression. We found that Bmara activated the transcription of *BmCHT10* by binding to the specific promoter region (Fig. 4c), in which no 5mC was found (Additional file 1: Table S2). The ara is a homeodomain-containing protein that acts as a transcriptional regulator to control biological processes like compound eye morphogenesis, imaginal disc-derived wing morphogenesis and vein specification [48, 49]. In our study, *Bmara* was up-regulated in the silkworm wings after 5-aza-dC treatment (Fig. 4b), its expression pattern was similar to *BmCHT10* (Fig. 2b). We found that Bmara enhanced *BmCHT10* transcription (Fig. 4d–f) by binding to the unmethylated the *BmCHT10* promoter region, as indicated by chromatin immunoprecipitation, EMSA and DNA pull-down assays (Fig. 5). In consequence, we proposed that DNA methylation inhibits *BmCHT10* transcription in the early pupal wing discs through Bmara repression. Indirect regulation of *BmCHT10* expression by DNA methylation is different from *BmCHSA*-*2b* expression, which is directly inhibited by methyl modification of the promoter, suggesting that DNA methylation affects wing development by directly or indirectly regulating the expression of wing development-related genes.

## Conclusions

By means of DNA methylation inhibitor (5-aza-dC) treatment and *BmDnmt1* RNAi to induce DNA de-methylation in *B. mori* at the early pupal stages, we demonstrated that DNA methylation inhibits *Bmara* expression, which in turn inhibits *BmCHT10* transcription to maintain chitin content for proper wing development.

## Materials and methods

### Insects and cell line

*Bombyx mori* larvae strain P50, obtained from the Research and Development Center of the Sericulture Research Institute of the Academy of Agricultural Sciences of Guangdong Province at China, were reared on fresh mulberry leaves at 27 °C with a 12 h light/12 h dark cycle. Under this condition for 6 days, the fifth instar silkworm larvae started wandering, followed by the beginning of larval–pupal transition. After pupation, the newly molted pupae (P0 stage) were transferred to a clean and dry paper box and reared at 27 °C for 7 days before enclosing into adult moth.

*Bombyx mori* Bm12 (DZNU-Bm-12) cell line, originally derived from the ovarian tissues [50], was cultured in Grace medium (Invitrogen, California, USA) supplemented with 10% fetal bovine serum (FBS) (Hyclone, Utah, USA) at 28 °C.

### 5-Aza-dC treatment experiment

For DNA methylation inhibitor treatment in silkworm pupae, 1 mg 5-aza-dC (5-aza-2′-deoxycytidine) (Sigma, California, USA) was dissolved in deionized distilled water (ddH_2_O) with a 10 μg/μL final concentration. Newly molted pupae (P0 stage) were injected into the abdomen with freshly prepared 5-aza-dC (20 μg per pupa) or ddH_2_O as control. Three replicates of about 30-40 pupae per replicate were carried out. In addition, for inhibitor 5-aza-dC treatment in the cultured *Bm*12 cells, the freshly prepared 5-aza-dC was diluted 10 times to 1 μg/μL final concentration, and 1 μg 5-aza-dC was applied to cells.

### Genomic DNA preparation and dot blot assay

For genomic DNA preparation, wing from silkworm pupae after 5-aza-dC or ddH_2_O treatment for 48 h and 72 h were homogenized with 600 μL digestive solution (5 mL 1 M Tris–HCl (pH 8.0), 4 mL 0.5 M EDTA (pH 8.0), 2 mL 5 M NaCl, 5 mL 10% (W/V) SDS, 150 μL 60 ng/μL Protease K), respectively, and the samples were digested overnight at 50 °C. Then, the genomic DNA was extracted with hydroxybenzene–chloroform–isopentanol, precipitated with absolute ethanol and washed with 75% ethanol.

For dot blot assay, genomic DNA samples were diluted into 100 ng/μL concentration and were digested by RNaseA (Promega) to rule out RNA contaminations. For each sample, 500 ng genomic DNA was denatured at 95 °C for 5 min and immediately cooled down on ice. Then, the genomic DNA was spotted on the nitrocellulose blotting membrane (PVDF, GE healthcare) and the membrane was dried on a heater, followed by UV-crosslinking for 10 min. The membrane was then blocked with 3% (w/v) BSA in TBST (20 mM Tris–HCl, 150 mM sodium chloride, 0.05% Tween-20, pH 7.4) for 2 h at room temperature, and incubated with rabbit anti-5mC antibody (diluted 1:1000; Abcam) overnight at 4 °C. After three washes in TBST for 10 min each, the membrane was incubated with a horse radish peroxidase (HRP)-linked secondary goat anti-rabbit IgG antibody (diluted 1:10,000; Dingguo Biotechnology) for 60 min at 37 °C. Meanwhile, the input genomic DNA samples were spotted on the PVDF membrane as mentioned above, and then directly stained with GoldView™ (Dingguo Biotechnology).

### Chitin staining assay

The pupal wing discs or adult wings from silkworm treated with 5-aza-dC or ddH_2_O were collected and fixed in 4% paraformaldehyde overnight. Pupal wing discs or adult wings were dehydrated by concentration gradients of alcohol and xylene, and embedded in paraffin. The embedded wing was sectioned (5 μm) using a microtome (Leica, Germany), affixed to slides, deparaffinized in xylene, and rehydrated with an ethanol gradient for chitin staining [51]. For histology structure observation, the wing slices were dyed with 0.01 mg/mL Fluorescent Brightener 28 (Sigma-Aldrich) for 90 s. After three washes in water for one min each, the tissue slices were counterstained with 0.01 mg/mL propidium iodide (Sigma-Aldrich) for 90 s and then rinsed three times in water for one min each. Finally, 50% glycerol was added to the tissue and then the slides were covered. Fluorescence signals were captured using a FV3000 confocal microscope (Olympus, Japan) at the excitation wavelengths of 365 nm and 535 nm.

### Chitin content assay

The pupal wing discs or adult wings from silkworm treated with 5-aza-dC or ddH_2_O were collected for chitin content assay. For each sample, four wings (two forewing and two hindwing wings) from one silkworm pupa were homogenized in 200 μL of 3% SDS (sodium dodecyl sulfate), incubated at 100 °C for 15 min and centrifuged at 1800*g* for 10 min at room temperature after cooling. After each pellet was washed with 500 μL ddH_2_O, it was resuspended in 300 μL of 120% KOH (w/v). To deacetylate chitins, the samples were incubated at 130 °C for 1 h followed by cooling them on ice for 5 min. Then, the sample was mixed with 800 μL of ice-cold 75% (v/v) ethanol, and incubated on ice for 15 min followed by centrifuging them at 1800*g* for 5 min at 4 °C. After each pellet containing insoluble chitosan (i.e., glucosamine polymer), then was washed with 500 μL of 40% (v/v) ice-cold ethanol and 500 μL of ice-cold ddH_2_O, the chitosan in each tube was re-suspended in 500 μL of ddH_2_O.

For chitin content assay, 500 μL of the chitosan solution was mixed with 50 μL of 10% NaNO_2_ (w/v) and 50 μL of 10% KHSO_4_ (w/v), and incubated at room temperature for 15 min followed by centrifuging them at 1800*g* for 15 min at 4 °C. Then, 60 μL of the supernatant of each sample was transferred to a new Eppendorf tube and mixed with 20 μL of NH_4_SO_3_NH_2_, and incubated at room temperature for 5 min. Each sample was added with 20 μL of freshly prepared MBTH (3-methyl-2-benzothiazolone hydrazone hydrochloride hydrate, Sigma-Aldrich), and incubated at 100 °C for 5 min. After cooling to room temperature, 100 μL of each sample was transferred to a well of a 96-wells plate and mixed with 20 μL FeCl_3_·6H_2_O. Absorbance of each sample was determined at a wavelength of 650 nm using a FlexStation3 microplate reader (Molecular Devices, USA) and the chitin content of each sample was described as a glucosamine equivalent according to a standard curve constructed using known concentration gradients of glucosamine (Sigma-Aldrich). All reported data were based on three biological replicates.

### RNA extraction and qRT-PCR

For RNA extraction, pupal wing discs, adult wings or *Bm*12 cells were homogenized with 1 mL of RNAiso Plus (TaKaRa, Dalian, China), and total RNA was extracted with chloroform, precipitated with isopropanol and washed with 75% ethanol. Pellets were suspended in the 30 μL RNase-free water and stored at − 80 °C. For cDNA synthesizing, 2 μg RNA was treated with Recombinant DNase I (RNase-free) (TaKaRa, Dalian, China) to remove the genome DNA and was then transcribed to cDNA using the First Strand cDNA Synthesis Kit (TaKaRa, Dalian, China) following the manufacturer’s instructions. The gene expression levels were analyzed using qRT-PCR with the Hieff™ qPCR SYBR Green Master Mix Kit (Yeasen, Guangzhou, China). The PCR conditions were as follows: initial denaturation at 95 °C for 30 s, followed by 40 cycles of 95 °C for 5 s and 60 °C for 31 s. The relative mRNA level of gene expression was normalized to the expression level of a housekeeping gene encoding ribosomal protein 49 (*rp49*) (GenBank accession no. 778453) and analyzed by the 2^−ΔΔCt^ method [52]. All reported data were based on three biological replicates and three technical replicates. All primers used for qRT-PCR listed in Additional file 1: Table S5.

### RNA-seq analysis

Total RNA was extracted from silkworm pupal wing discs after 5-aza-dC or ddH_2_O treatment in newly molted pupae for 48 h using RNAiso Plus (TaKaRa, Dalian, China) as described above. RNA-seq was done on an Illumina HiSeq™ 2500 sequencing platform by the Biomarker Technologies and paired-reads with an average length of approximately 200 nt were generated. The clean reads that were filtered from the raw reads were used for mapping to the reference *B. mori* genomes. After mapping, the assembled transcripts were generated, and gene expression level of each gene was normalized using FPKM values (fragments per kilobase of exon per million fragments mapped) by the Cufflinks software [53]. All reported data were based on three biological replicates.

### Expression, purification of recombinant proteins and polyclonal antibody preparation

The ORF (open reading frame) of select genes were amplified by PCR. The cDNAs were subcloned into the pPET-28a/32a vector infused with a 6× His to generate the recombinant expression vectors, respectively. The recombinant proteins were expressed in *Escherichia coli* cells (BL21). All primers used for PCR listed in Additional file 1: Table S5.

For purification of proteins with His tag, the transformed *E. coli* cells were collected by centrifugation and re-suspended in the binding buffer (0.5 M NaCl, 20 mM Tris–HCl, 5 mM Imidazole, pH 7.9 and 1 mM PMSF). The suspension was centrifuged after being lysed by sonication and then purified with Ni-chelating affinity chromatography using the His-Bind^®^ 12 Kit according to the manufacturer’s protocol (Novagen, Wisconsin, USA).

Mouse anti-BmCHT10 and mouse anti-Bmara antibodies were prepared, respectively. In brief, mouse strain BALB/c males, obtained from Guangdong Medical Laboratory Animal Center (GDMLAC), were used for polyclonal antibody preparation by intraperitoneal injection. Mixed the antigens (purified recombinant proteins) with an equal volume of the Freund’s Adjuvant Complete or Incomplete (Sigma-Aldrich) was mixed to form an emulsion. The Complete Freund’s Adjuvant was used for the initial injections and the Incomplete Freund’s Adjuvant was used in the later injections for the boosts. After four times injections with a 200 μL of emulsion (once a week), the mouse serums were isolated and incubated at 37 °C for 2 h. After gentle centrifugation, the supernatants were stored at − 80 °C and used as the primary antibody in the following Western blot analysis and immunohistochemistry experiment.

### Western blotting

The pupal wing discs or *Bm*12 cells were homogenized in Cell Lysis Buffer (Beyotime, Shanghai, China). For Western blot, 40-100 μg proteins extracted from tissues or *Bm*12 cells were denatured and then separated in 12% SDS-PAGE gel, followed by transferring to a nitrocellulose blotting membrane (GE healthcare). The membrane was blocked with 3% (w/v) BSA in TBST for 2 h at room temperature, followed by hybridization overnight at 4 °C in TBST containing 1% BSA and primary antibody (diluted 1:1000, polyclonal antibodies as described above). The secondary antibody was a horse-radish peroxidase (HRP)-conjugated goat anti-rabbit IgG (diluted 1:10,000, Dingguo Biotechnology). Antibody against tubulin (diluted 1:5000, Dingguo Biotechnology) was used to verify equal loading of the proteins on the gel.

### Construction of the reporter luciferase vector

Genomic DNA was extracted from the *Bm*12 cells. The promoter upstream the ATG of *BmCHT10* was amplified by PCR according the sequence of its genome DNA sequence (Gene ID: 101736080) and cloned into pMD-18T vectors (TaKaRa, Dalian, China). After the digestion by double restriction enzymes, Sma I and BgI II, the promoter fragments were cloned into the luciferase reporter plasmid, pGL3-basic vector (Promega, Madison, USA). The primers used for constructing the vectors are listed in Additional file 1: Table S5.

### Cell culture, transfection and transcriptional activity assay

*Bm*12 cells at their logarithmic growth phase were inoculated in culture media in 12- or 24-well culture plates (Corning, New York, NY, USA) and cultured for 12 h. Cell transfection and co-transfection were conducted when the cells were at approximately 80% density. Plasmid DNAs were mixed with Fugene HD transfection reagent (Promega, Madison, USA) and added to cells in each well of 12- or 24-well culture plates with Grace medium (Invitrogen). To normalize the firefly luciferase activity, the renilla luciferase vector, pRL-SV40, was co-transfected with each of the pGL3-derived vectors containing tested promoters. The cells were cultured for additional 48 h at 28 °C, followed by the luciferase activity assay, protein or RNA isolation.

For luciferase activity measurement, the cells were washed twice with filtered PBS and then lysed in 100 μL Passive Lysis Buffer (Promega, Madison, WI, USA). The samples were centrifuged at 800*g* for 5 min at room temperature. The supernatant was used to analyze the luciferase activity using the Dual- Luciferase Assay System according to the manufacturer’s protocol with a luminometer (IBA7300, Veritas, Turner Biosystems). The luciferase activity was normalized to the renilla luciferase activity. All assays included three biological replications and three technical replicates. The luciferase activity was represented as mean ± standard error (SE).

### RNA interference (RNAi)

For RNAi in the *Bm*12 cell line, a 400–600 bp unique fragment in the ORF of target genes was chosen as a template for synthesizing gene-specific dsRNA using the T7 RiboMAXTM Express RNAi System (Promega, Wisconsin, USA). dsRNA (1 μg) was used to transfect the *Bm*12 cells with 4 μl Fugene HD transfection reagent in the Opti-MEM Reduced Serum Medium. The cells were collected 48 h after transfection. All assays included three biological replications. The sequences of primers are listed in Additional file 1: Table S5.

### Immunohistochemistry

The newly dissected silkworm pupal wing discs or adult wings were fixed in 4% paraformaldehyde for 30 min at room temperature. For BmCHT10 protein and chitin staining, pupal wing discs or adult wings were blocked in PBS containing 5% BSA and 0.5% Triton-X (PBT) for 1–2 h, and then incubated with the primary antibody (mouse anti-BmCHT10, diluted 1:200) at 4 °C overnight. After being washed three times for 10 min each in 0.2% PBT, the samples were then incubated with Alexa Fluor™ 488 goat anti-mouse IgG (diluted 1:200; Invitrogen) for 2 h. Fluorescent Brightener 28 (Sigma-Aldrich) was added to stain chitin. The wings stained with anti-BmCHT10 and Fluorescent Brightener 28 were observed and imaged using a FV3000 confocal microscope (Olympus).

For 5mC staining, the fixed pupal wing discs or adult wings were first incubated with 2 M HCl solution for 20 min and then neutralized with 100 mM Tris–HCl (pH 8.5) for 10 min at room temperature. After blocking, the pupal wing discs or adult wings were incubated with the primary antibody (rabbit anti-5mC, diluted 1:1000, Abcam) at 4 °C overnight. After 3 washes, the samples were incubated at room temperature for 2 h with the goat anti-rabbit IgG secondary antibody conjugated with Alexa Fluor™ 594 (diluted 1:200, Invitrogen). The nuclei were stained with DAPI (Beyotime, Shanghai, China) for 20 min. Then the wings stained with anti-5mC and DAPI were observed and imaged using a FV3000 confocal microscope (Olympus).

### BS-seq analysis

Genomic DNA of *B. mori* was extracted from pupal wing discs as described above. After treated with bisulfite, unmethylated cytosines were converted into uracil using MethylDetectorTM (Active Motif, Carlsbad, CA, USA), whereas methylated cytosines remain unchanged. Polymerase chain reaction (PCR) was then performed and PCR products were cloned into pMD18-T vector for following sequencing. By aligning with the sequence of unconverted gDNA using DNAMAN software (Lynnon Biosoft), a single-base-resolution DNA methylation distribution can be quantified. All assays included three biological replications. The results of BS-seq are listed in Additional file 1: Table S2.

### Nuclear protein preparation and DNA pull-down

Nuclear proteins were extracted from *Bm*12 cells according to the instruction of NE-PER Nuclear and Cytoplasmic Extraction Kit (Thermo Scientific, Waltham, USA). For DNA pull-down assay, the oligonucleotides conjugated with biotin at 5′ end were synthesized by Qingke Biotechnology, and the single-stranded oligo-probes were heated at 95 °C for 10 min and then slowly cooled to room temperature to obtain the double-stranded probes. Then, the oligo-probes were linked to the streptavidin-coated beads. To minimize non-specific interactions, the oligo-bead complexes were incubated for 30 min with a blocking buffer (2.5 mg/mL albumin from bovine serum (BSA), 10 mM HEPES pH 7.6, 10 mM glutamate potassium, 2.5 mM DTT, 10 mM magnesium acetate, 5 mM EGTA, 3.5% glycerol with 0.003% NP-40 and 5 mg/mL polyvinylpyrrolidone). Immobilized double-stranded probes were incubated with 20 μg of nuclear extract for 4 h at 4 °C with constant rotation in a 400 μL of protein binding buffer (10 mM HEPES pH 7.6, 100 mM glutamate potassium, 80 mM potassium chloride, 2.5 mM DTT, 10 mM magnesium acetate, 5 mM EGTA, 3.5% glycerine with 0.001% NP-40). Protein-DNA complexes were then washed three times with a wash buffer (10 mM HEPES pH 7.6, 100 mM glutamate potassium, 2.5 mM DTT, 10 mM magnesium acetate, 5 mM EGTA, 3.5% glycerol, 0.5 mg/mL BSA, 0.05% NP-40). Proteins bound to the probe were eluted with 20 μL of a denaturing Laemmli sample loading buffer (50 mM Tris–HCl, 100 mM DTT, 2% SDS, 0.1% bromophenol blue, 10% glycerol) at 37 °C for 15 min. The target proteins in the supernatant were identified by Western blot with anti-FLAG antibody (#14793, Cell Signaling Technology, MA, USA) at 1:2000 dilution.

### Electrophoretic mobility shift assay (EMSA)

The recombinant Bmara protein was prepared as described above. EMSA was conducted using the LightShift Chemiluminescent EMSA Kit (Thermo Scientific). The oligonucleotide probes conjugated with biotin at 5′ end were heated at 95 °C for 10 min and then slowly cooled to room temperature. Binding assays were performed according to the manufacture’s protocol. Briefly, the recombinant Bmara proteins were incubated for 20 min at room temperature with 20 μL binding buffer containing 50 ng of poly (dI-dC), 2.5% glycerol, 0.05% NP-40, 50 mM potassium chloride, 5 mM magnesium chloride, 4 mM EDTA and 20 fmol of a biotinylated end-labeled double-stranded probe. Different concentrations of cold probes (unlabeled) were added into the binding mixture as competitors. Polyacrylamide gels (6%) were run at 100 volts for 1.5 h on ice. After electrophoresis, the proteins were blotted onto positively charged nylon membranes (Hybond Nþ; Amersham Biosciences) and the bands were visualized using the EMSA Kit according to the manufacturer’s protocol.

### Chromatin immunoprecipitation (ChIP)

ChIP was performed in the *Bm*12 cells following the instruction of Pierce™ Magnetic ChIP Kit (Thermo Scientific). Briefly, approximately 4 × 10^6^ cells were set up, cross-linked with 1% formaldehyde for 10 min after transfected with overexpression vectors of Bmara-FLAG for 48 h, and then de-cross-linked with glycine. The cells were broken up with extraction buffer containing protease/phosphatase inhibitors. The nuclei were treated with the MNase diluted in MNase Digestion Buffer for 15 min at 37 °C, and the nuclei were released from the cells using several ultrasonic pulses and 20 s ice-cold interval. The protein-DNA complexes were immunoprecipitated using anti-FLAG antibody (#14793, Cell Signaling Technology, MA, USA) or normal rabbit IgG (as a control) (Thermo Fisher Scientific, Massachusetts, USA) for 8 h at 4 °C with constant mixing in a Mini Rotating Incubator (Qilinbeier, Haimen, China), and then enriched by Protein A/G Magnetic Beads for 2 h at 4 °C with mixing before being reversely cross-linked at 65 °C for 30 min with vigorous rotation in thermomixer comfort (Eppendorf, Hamburg, Germany). DNA was purified using the column method (Thermo Scientific), and detected by reverse transcription PCR. All assays included three biological replications.

### Bioinformatics analysis

The amino acid sequences were downloaded from the NCBI protein database. The conserved domain of the protein amino acid sequences was predicted using SMART [54]. The multiple sequence alignment of the protein amino acid sequences was aligned with Clustal Omega [55]. The results of conserved domain prediction and multiple sequence alignment were listed in Additional file 1: Figure S1.

The *cis*-regulation elements (CRE) were predicted with the JASPAR 2020 [46]. The protein molecular weight of the candidate transcription factors was analyzed with The Sequence Manipulation Suite [56]. The results of CRE prediction and protein molecular weight analysis were listed in Additional file 1: Table S3.

### Statistical analysis

Data are presented as mean ± SE. *p* values for the purpose of group comparisons were calculated using student’s *t* test (*p < 0.05, **p < 0.01, ***p < 0.001).

## Supplementary information

**Additional file 1: Figure S1.** Amino acid sequence and conserved domain analysis of CHT10 proteins in diverse insect species. **Figure S2.** RT-PCR (above) and qRT-PCR (below) analyses of *BmDnmt1* mRNA levels post *BmDnmt1* RNAi. The *Bm*12 cells were transfected with *dsBmDnmt1* or *dsgfp* (control). **Figure S3.** Effects of *Bmaraucan, Bmcaupolican* or *Bmhomothorax* RNAi on the promoter activity of the − 250 to − 1 nt of *BmCHT10* promoter in the *Bm*12 cells. **Figure S4.** The green fluorescence shows the similar transfection efficiency and expression of EGFP or Bmara at 48 h post transfection. The *Bm*12 cells transfected with EGFP-N1 vector was used as a control. **Figure S5.** RT-PCR (above) and qRT-PCR (below) analyses of *Bmara* mRNA levels post *Bmara* RNAi. The *Bm*12 cells were transfected with *dsBmara* or *dsgfp* (control).**Table S1.** Differentially expressed genes (DEGs) in chitin metabolism and wing cuticle protein in 5-aza-dC-treated 3-day-old pupal wing discs. **Table S2.** BS-seq analysis of methylation rate of C sites in the − 250 to − 225 nt region of the *BmCHT10* promoter. **Table S3.** *Cis*-regulation elements (CRE) prediction of the − 250 to − 225 nt fragment in the *BmCHT10* promoter and the protein molecular weight (MW) analysis of the predicted CRE-bound transcription factors (TFs). **Table S4.** *Cis*-regulation elements (CREs) in the − 250 to − 225 fragment of the *BmCHT10* promoter. **Table S5.** List of primers used in this study.

## Data Availability

The raw Illumina sequencing data from RNA-seq are deposited in SRA with the accession number SRP234898 at NCBI.
