# Supplementary material for: DNA methylation suppresses chitin degradation and promotes the wing development by inhibiting Bmara-mediated chitinase expression in the silkworm, Bombyx mori
Source: Epigenetics Chromatin. 2020 Sep 4;13:34. doi: 10.1186/s13072-020-00356-6 (PMC7472703; doi:10.1186/s13072-020-00356-6)
Supplement: Supplementary file 1 — Additional file 1: Figure S1. Amino acid sequence and conserved domain analysis of CHT10 proteins in diverse insect species. Figure S2. RT-PCR (above) and qRT-PCR (below) analyses of BmDnmt1 mRNA levels post BmDnmt1 RNAi. The Bm12 cells were transfected with dsBmDnmt1 or dsgfp (control). Figure S3. Effects of Bmaraucan, Bmcaupolican or Bmhomothorax RNAi on the promoter activity of the − 250 to − 1 nt of BmCHT10 promoter in the Bm12 cells. Figure S4. The green fluorescence shows the similar transfection efficiency and expression of EGFP or Bmara at 48 h post transfection. The Bm12 cells transfected with EGFP-N1 vector was used as a control. Figure S5. RT-PCR (above) and qRT-PCR (below) analyses of Bmara mRNA levels post Bmara RNAi. The Bm12 cells were transfected with dsBmara or dsgfp (control).Table S1. Differentially expressed genes (DEGs) in chitin metabolism and wing cuticle protein in 5-aza-dC-treated 3-day-old pupal wing discs. Table S2. BS-seq analysis of methylation rate of C sites in the − 250 to − 225 nt region of the BmCHT10 promoter. Table S3. Cis-regulation elements (CRE) prediction of the − 250 to − 225 nt fragment in the BmCHT10 promoter and the protein molecular weight (MW) analysis of the predicted CRE-bound transcription factors (TFs). Table S4. Cis-regulation elements (CREs) in the − 250 to − 225 fragment of the BmCHT10 promoter. Table S5. List of primers used in this study. [file 13072_2020_356_MOESM1_ESM.pdf]

Additional file

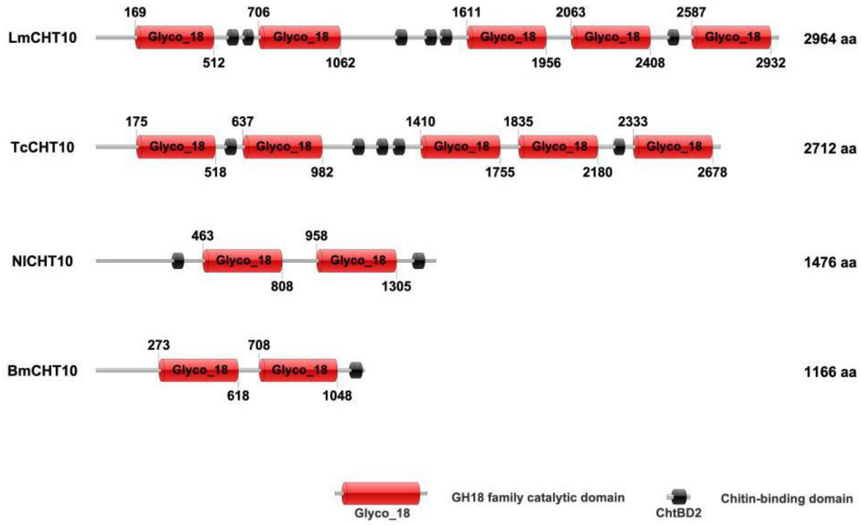

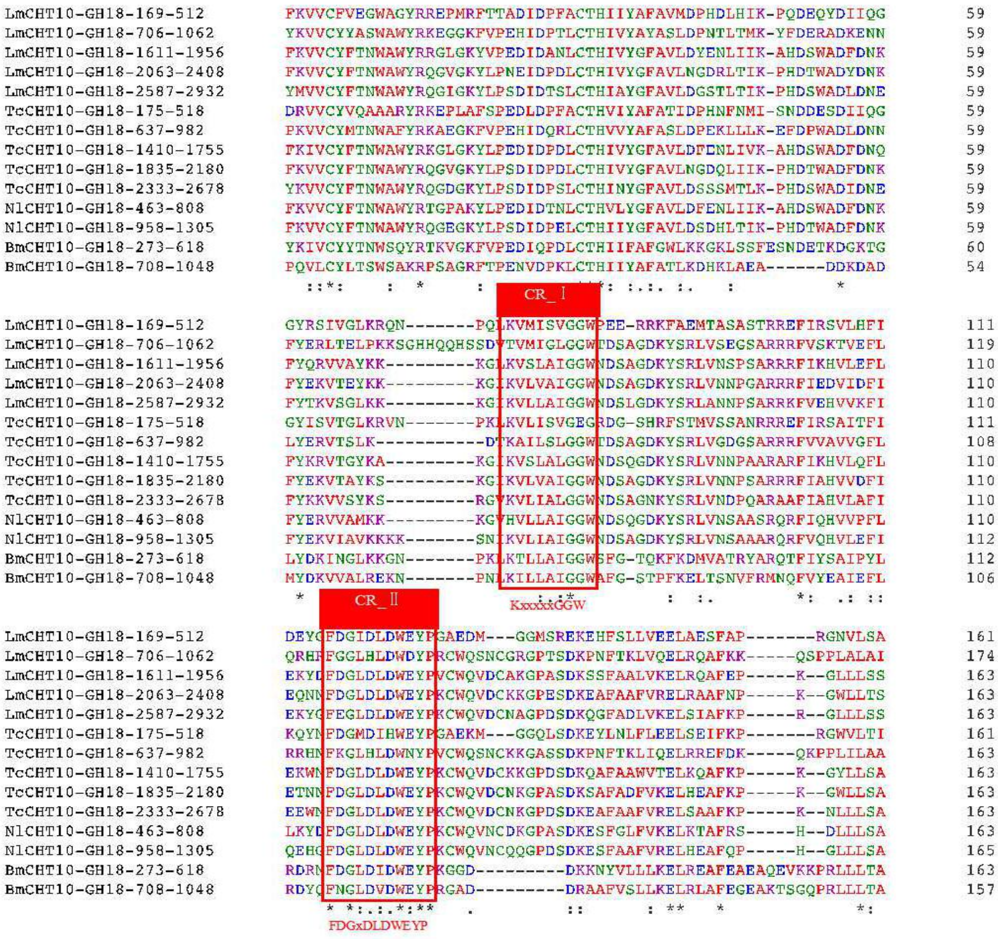

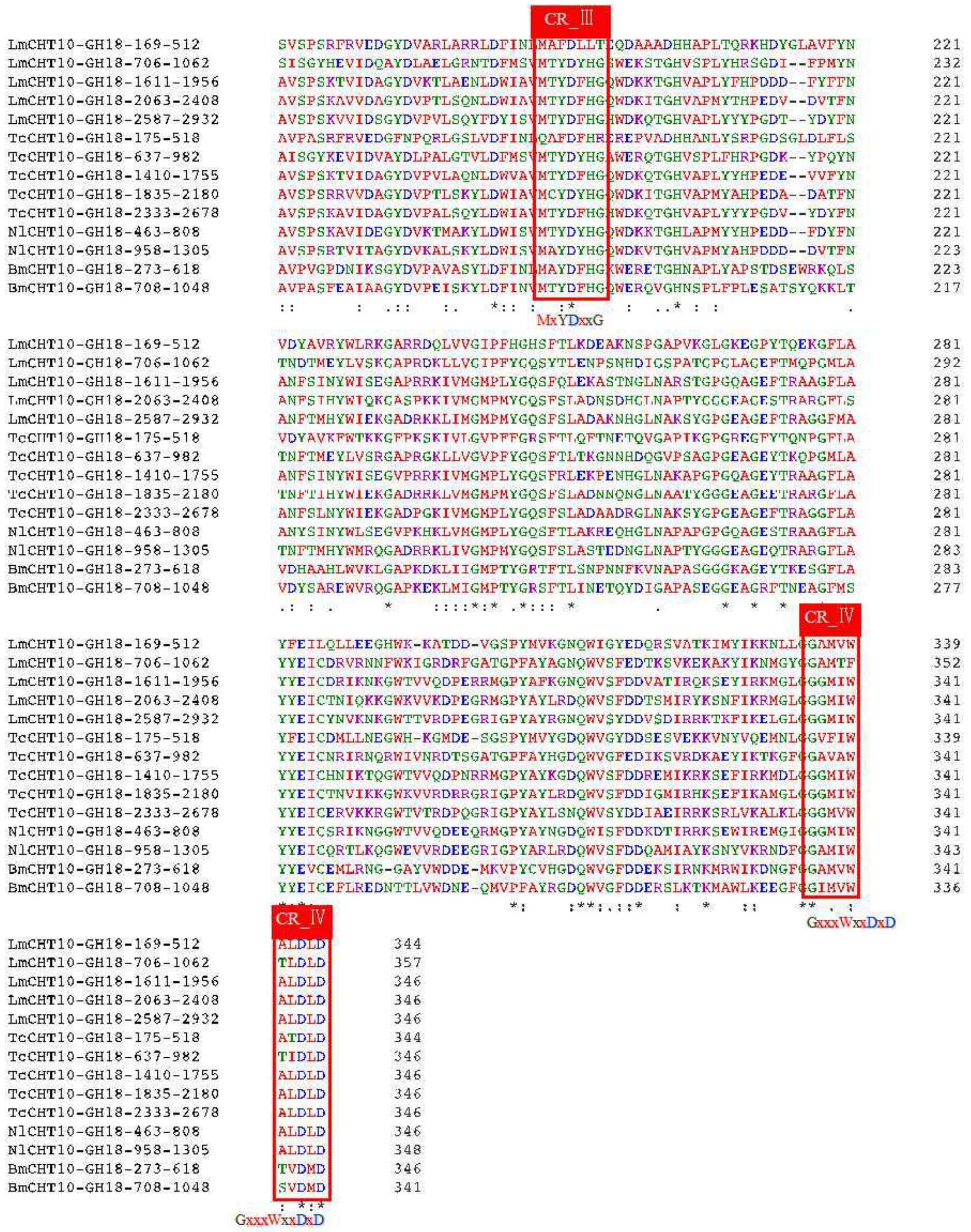

Figure S1. Amino acid sequence and conserved domain analyses of CHT10 proteins in diverse insect species. Schematic diagram of the CHT10 amino acid domains in *Locusta migratoria* (AMT75074.1), *Tribolium castaneum* (XP_008198138.1), *Nilaparvata lugens* (XP_022199698.1) and *Bombyx mori* (XP_021203926.1) (Top). The amino acid sequences of chitinase 10 (CHT10) proteins in the insect species mentioned above contains two conserved domain, glycoside hydrolase family 18 (GH18 family) catalytic domain (Glyco_18) and Chitin-binding domain (ChtBD2).

Multiple amino acid sequences alignment of Glyco_18 domains of the CHT10 proteins in different insects. Boxed regions are the four conserved regions (CRs) presented by the sequences KxxxxxGGW, FDGxDLDWEYP, MxYDxxG and GxxxWxxDxDD (x represents a non- specific amino acid) (bottom).

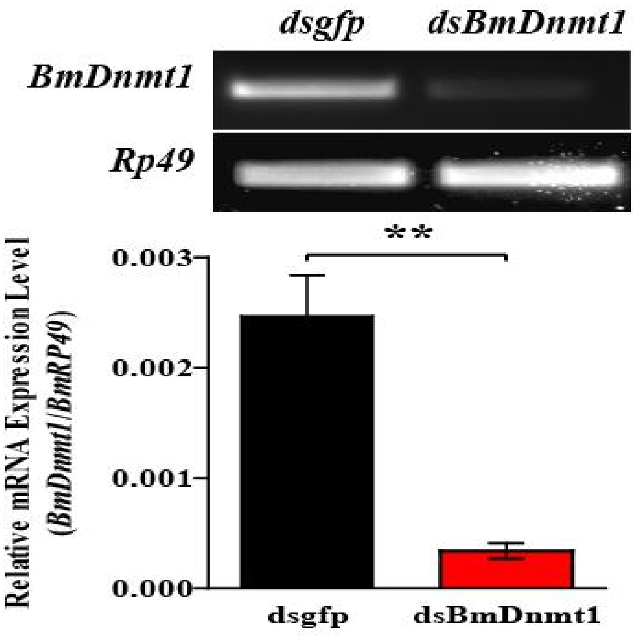

Figure S2. RT-PCR (above) and qRT-PCR (below) analyses of *BmDnmt1* mRNA levels post *BmDnmt1* RNAi. The Bm12 cells were transfected with *dsBmDnmt1* or *dsgfp* (control). Each data point is the mean ± SE of three independent assays. For the *t* test: $p<0.05$ (*) or $p<0.01$(**).

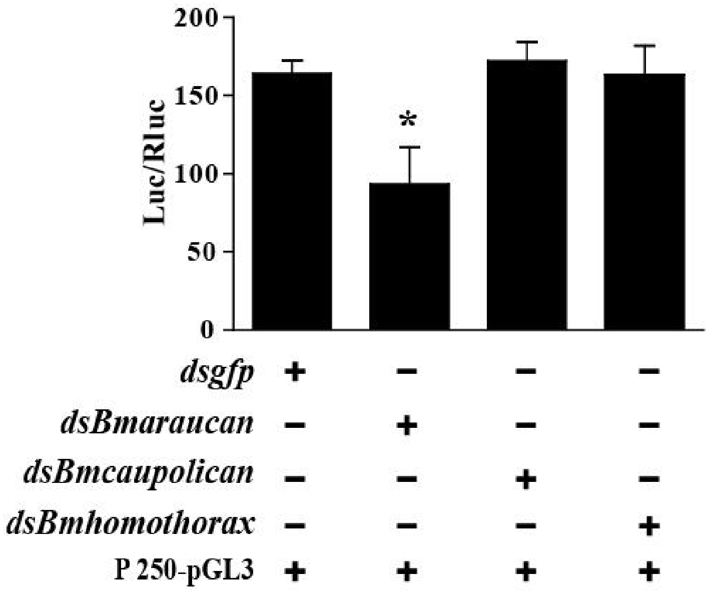

Figure S3. Effects of *Bmaraucan, Bmcaupolican* or *Bmhomothorax* RNAi on the promoter activity of the -250 - -1 nt of *BmCHT10* promoter in the Bm12 cells. *dsgfp* or *dsBmDnmt1* co-transfected with the vector including -250 - -1 nt fragment for the determination of luciferase activity. Each data point is the mean ± SE of three independent assays. For the t test: p<0.05 (*).

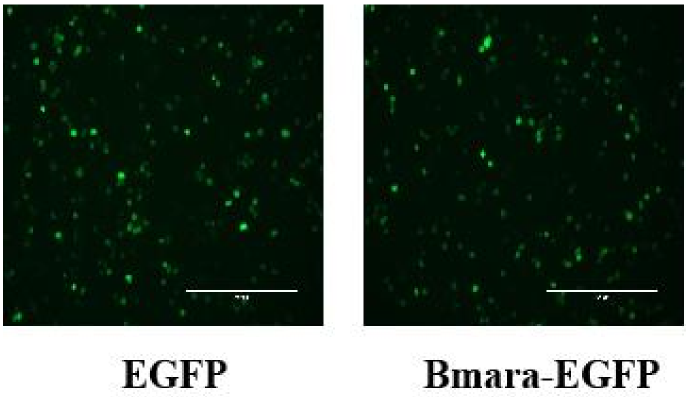

Figure S4. The green fluorescence shows the similar transfection efficiency and expression of EGFP or Bmara at 48 h post transfection. The Bm12 cells transfected

with EGFP-N1 vector as a control.

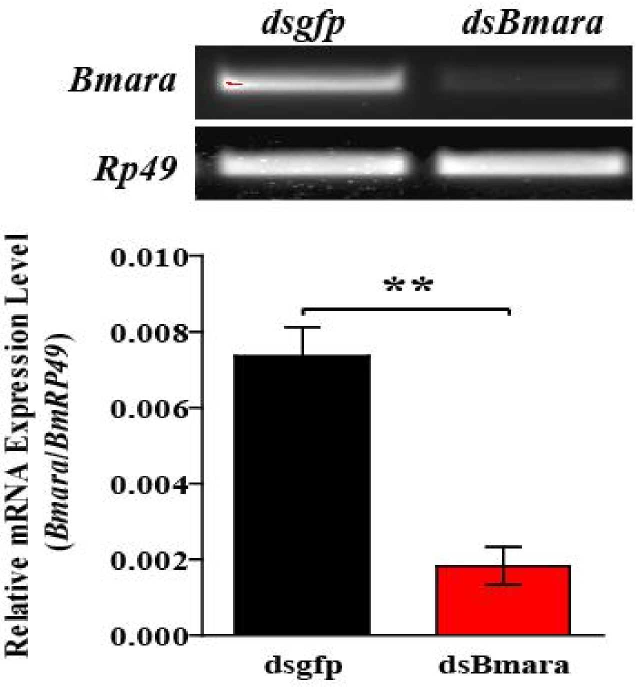

Figure S5. RT-PCR (above) and qRT-PCR (below) analyses of *Bmara* mRNA levels post *Bmara* RNAi. The *Bm*12 cells were transfected with *dsBmara* or *dsgfp* (control). Each data point is the mean ± SE of three independent assays. For the *t* test: $p<0.05$ (*) or $p<0.01$(**).

Table S1. Differentially expressed genes (DEGs) on chitin metabolism and Wing cuticle protein after 5-aza-dC treatment in P3 stage silkworm wings

Gene	5-aza-dC _48 h RPKM	ddH_2O _48 h RPKM	nr_annotation
BGIBMGA000275	5.15165	0.964013	cuticular protein RR-2 motif 89 precursor [Bombyx mori]
BGIBMGA012596	2.070392	0.192194	cuticular protein RR-1 motif 17 precursor [Bombyx mori]
BGIBMGA012602	0.851775	0.084943	cuticular protein RR-1 motif 12 precursor [Bombyx mori]
BGIBMGA012605	3.32339	0.935883	cuticular protein RR-1 motif 15 precursor [Bombyx mori]
BGIBMGA000338	22.2729	3.56065	cuticular protein RR-1 motif 32 precursor [Bombyx mori]
BGIBMGA000333	20.5609	1.11285	larval cuticle protein LCP-22 precursor [Bombyx mori]
BGIBMGA000334	9.19737	2.80923	cuticular protein RR-1 motif 37 precursor [Bombyx mori]
BGIBMGA006874	4.587355	0.697745	chitinase-related protein 1 [Danaus plexippus]
BGIBMGA005539	2.800065	0.450118	PREDICTED: probable chitinase 10-like isoform X2 [*Bombyx mori*]
*Bombyx_mori*_newGene_5757	4.85778	1.1948	PREDICTED: probable chitinase 10-like isoform X2 [*Bombyx mori*]

Table S2. BS-seq analysis of methylation level of C sites on upstream 250-225 nt in the *BmCHT10* promoter.

Strand(+/-)	Position	Methylation type	Adjacent sequence	Methylation rate(%)
+	upstream 224 nt	CG	CACGG	0
+	upstream 226 nt	CHH	AACAC	0
+	upstream 229 nt	CHH	TTCAA	0
+	upstream 233 nt	CHH	GTCTT	0
+	upstream 237 nt	CHG	GGCTG	0
+	upstream 240 nt	CG	CACGG	0
+	upstream 242 nt	CHH	TCCAC	0
+	upstream 243 nt	CHH	GTCCA	0
+	upstream 247 nt	CHG	TTCAG	0
–	upstream 222 nt	CHG	GCCTG	0
–	upstream 223 nt	CHH	TGCCT	0
–	upstream 235 nt	CHG	GACAG	0
–	upstream 238 nt	CG	GCCGA	0
–	upstream 239 nt	CHG	TGCCG	0
–	upstream 245 nt	CHG	GTCAG	0

Table S3. *cis*-regulation elements (CRE) prediction of the -250- -225 nt fragments in the *BmCHT10* promoter and the protein molecular weight (MW) analysis of the predicted CRE-binding transcription factors (TFs).

Strand	Start	End	Predicted sequence	Name of CREs	GenBank accession no.	Protein MW (kDa)
+	19	24	TTCAAC	cut	101736914	132.29
**+**	**21**	**25**	**CAACA**	**araucan**	**101745000**	**51.13**
+	21	25	CAACA	caupolican	101740424	45.8
-	13	18	AGACAG	achintya	100862767	37.35
-	13	18	AGACAG	homothorax	101737806	46.92
**-**	**14**	**18**	**AGACA**	**araucan**	**101745000**	**51.13**
-	14	18	AGACA	caupolican	101740424	45.8
-	17	22	TGAAAG	achintya	100862767	37.35
-	17	22	TGAAAG	homothorax	101737806	46.92
-	18	23	TTGAAA	cut	101736914	132.29

Table S4. *cis*-regulation elements (CREs) in the -250- -225 nt fragment of the *BmCHT10* promoter.

Name of CREs	Match Total	-1100- -900 nt	-900- -700 nt	-400- -300 nt	-250- -225 nt
**araucan**	**18**	**5**	**8**	**3**	**2**
achintya	4	0	1	2	2
cut	2	0	0	0	2
caupolican	2	0	0	0	2
homothorax	2	0	0	0	2

Table S5. List of primers used in this study.

Primer	Sequence (5'-3')
**Primers for qRT-PCR**	
BmCHT10-qF	TACCGGACGAAGGTCGGGAAGT
BmCHT10-qR	TTCAGCCAGCCGAAAGCGAAGA
RP49-qF	CAGGCGGTTCAAGGGTCAATAC
RP49-qR	TACGGAATCCATTTGGGAGCAT
Bmara-qF	ACCCTTACCCCACGAAAGGGGA
Bmara-qR	AGTCGTCGGCGTGCGTTCG
BmDnmt1-qF	CTCTGCGAGCTTTGTTGGACATG
BmDnmt1-qR	CGCTGCCGCCAATATGATCAAC
**Primers for promoter reporter construction (*BmCHT10*)**	
CHT10-P1100-F:	cccggg TCGAGCCTAACGAATTAATTTCA
CHT10-P900-F:	cccggg AGAAAAATTGGTACCCGCCTG
CHT10-P700-F	cccggg TGCCGTGAAGCAGTAGTGCG
CHT10-P500-F	cccggg CACAATTTAACTGAAGCAAAAACTTC
CHT10-P400-F	cccggg AATAGTGTTTCGCGCCATAGC
CHT10-P300-F	cccggg GCGTACGCGCATTCTGTATTC
CHT10-P200-F	cccggg ATACGCAGATTCGAAATTC
CHT10-P150-F	cccggg TTATAATTTTTTTGTTTTGATTGTTTATT
CHT10-P100-F	cccggg TTTTGTGAACGACTGTACTTACTGTGC

CHT10-P275-F	cccggg GACGGGCGCCGACGACTT
CHT10-P250-F	cccggg TTCAGTCCACGGCTGTCTTTC
CHT10-P225-F	cccggg CGGACAGGCGAGAGGACAAAT
CHT10-P-R	agatct CGCACCACAAACACAGTACCG
**Primers for protein overexpression in the Bm12 cells**	
Bmara-GFP-F	ggtaccGATGGCAGCGTACGCGCAATTTGGT
Bmara-GFP-R	ggatccTTAGAAGGCCGCAGAGCCTTCGT
Bmara-Flag-R	ggatccTTACTTGTCATCGTCGTCCTTGTAATCGAAG GCCGCAGAGCCTTCGT
**Primers for protein expression in bacterial cells**	
BmCHT10-CDS-1318-F	ggatcc GATGAGAAATCAATCAGGAACAAAAT
BmCHT10-CDS-2402-R	gaattc TTACCTTCCGTACGTGGGCAT
BmCHT10-CDS-1934-F	ggatcc TCGAACGTGTTCCGCATGA
BmCHT10-CDS-2850-R	gaattc TTAGCACGTCACCTCAGTGGGG
Bmara-F	ggatcc ATGGCAGCGTACGCGCAATTTGGT
Bmara-R	gaattc TTAGAAGGCCGCAGAGCCTTCGT
**Primers for RNAi**	
RNAi Bmara-F	CGTGGCTCAACGAACACAAG
RNAi Bmara-R	TACCGTCAATTGGTGCTGCT
RNAi Bmara-T7-F	taatacgactcactataggCGTGGCTCAACGAACACAAG
RNAi Bmara-T7-R	taatacgactcactataggTACCGTCAATTGGTGCTGCT
RNAi GFP-F	TACGGCGTGCAGTGCTTCAGCC

RNAi GFP-R	GTGCTCAGGTAGTGGTTGTCGG
RNAi GFP-T7-F	taatacgactcactataggTACGGCGTGCAGTGCTTCAGCC
RNAi GFP-T7-R	taatacgactcactataggGTGCTCAGGTAGTGGTTGTCGG
RNAi Dnmt1-F	ATCAAGCTGGAGTTGCAGAATG
RNAi Dnmt1-R	TTAAAGGCCACAAAGTTACGAA
RNAi Dnmt1-T7-F	taatacgactcactataggATCAAGCTGGAGTTGCAGAATG
RNAi Dnmt1-T7-R	taatacgactcactataggTTAAAGGCCACAAAGTTACGAA
RNAi Bmcaup-F	TCAAAGCATGGCTCAACGAG
RNAi Bmcaup-R	AGGCGACTCACCGTCTTTG
RNAi Bmcaup-T7-F	taatacgactcactataggTCAAAGCATGGCTCAACGAG
RNAi Bmcaup-T7-R	taatacgactcactataggAGGCGACTCACCGTCTTTG
RNAi Bmhth-F	CGTCAGACCTCCTTCGTCAT
RNAi Bmhth-R	TTGGTTGGACTATCCTACGTCTC
RNAi Bmhth-T7-F	taatacgactcactataggCGTCAGACCTCCTTCGTCAT
RNAi Bmhth-T7-R	taatacgactcactataggTTGGTTGGACTATCCTACGTCTC
**Primers for EMSA and DNA pull-down**	
Wt CRE-F	TTCAGTCCACGGCTGTCTTTCAACACGGA
Wt CRE-R	TCCGTGTTGAAAGACAGCCGTGGACTGAA
Mut-CRE-F	TTCAGTCCACGGCgtgacTTtcgacCGGA
Mut-CRE-R	TCCGgtcgaAAgtcacGCCGTGGACTGAA
**Primers for ChIP**	
ChIP CRE-F	CGTACGCGCATTCTGTATTCG

ChIP CRE-R	TTTTTGAATTTCGAATCTGCGTAT
